# Electrochemical Determination of Metronidazole in Tablet Samples Using Carbon Paste Electrode

**DOI:** 10.1155/2016/3612943

**Published:** 2016-03-29

**Authors:** Yosef Nikodimos, Meareg Amare

**Affiliations:** Department of Chemistry, Bahir Dar University, P.O. Box 79, Bahr Dar, Ethiopia

## Abstract

Cyclic voltammetric investigation of metronidazole at carbon paste electrode revealed an irreversible reduction peak centered at about −0.4 V. Observed peak potential shift with pH in the range 2.0 to 8.5 indicated the involvement of protons during the reduction of metronidazole, whereas the peak potential shift with scan rate in the range 10–250 mV/s confirmed the irreversibility of the reduction reaction. A better correlation coefficient for the dependence of peak current on the scan rate than on the square root of scan rate indicated an adsorption controlled kinetics. Under the optimized method and solution parameters, an excellent linearity between the reductive peak current and the concentration of metronidazole was observed in the concentration range 1.0 × 10^−6^ to 5.0 × 10^−4^ M with a correlation coefficient, method detection limit (based on *s* = 3*σ*), and limit of quantification of 0.999, 2.97 × 10^−7^ M and 9.91 × 10^−7^ M, respectively. Good recovery results for spiked metronidazole in tablet samples and selective determination of metronidazole in tablet formulations in the presence of selected potential interferents such as rabeprazole, omeprazole, and tinidazole confirmed the potential applicability of the developed method for the determination of metronidazole in real samples like pharmaceutical tablets.

## 1. Introduction

Metronidazole (*2-methyl-5-nitroimidazole-1-ethanol*) belongs to a group of nitroimidazole drugs used in therapeutics mainly in the treatment of infections caused by susceptible organisms, particularly anaerobic bacteria (*Bacteroides*,* Fusobacterium*,* Campylobacterium*, and* Clostridium*) and protozoa (*Trichomonas*,* Treponema*, and* Histomonas*) [1, 2]. It can kill or inhibit the majority of anaerobic bacteria when the metronidazole concentration in serum is in the range of 2 to 8 mg/mL [3].

Due to its antimicrobial activity, rapid bacterial killing, good tissue penetration, low cost, and limited adverse ejects, metronidazole (MTZ) is the drug of choice for prophylaxis and treatment of patients with Crohn's disease and ulcerative colitis to prevent infectious complications [4, 5].

The pharmacokinetic and pharmacodynamic properties of the drug are favorable, and it is available as oral, intravenous, vaginal, and topical formulations. After oral administration, metronidazole is well absorbed, and its peak plasma concentrations occur 1-2 h after administration [1, 4].

In accordance with the international guidelines, metronidazole is also a component of multidrug regimens (e.g., in combination with omeprazole, rabeprazole, and amoxicillin) for therapy of* Helicobacter pylori* infections, which is a major cause of gastritis and a risk factor for stomach cancer [4, 6].

However, high doses and long-term systemic treatment with metronidazole are associated with the development of leucopenia, neutropenia, increased risk of peripheral neuropathy, and toxicity of the central nervous system. In clinical studies, where high doses of metronidazole were used during radiation treatment for cancer, an overdose was believed to increase the risk of seizures or nerve problems in the hands and feet [7]. The side effects may vary from patient to patient depending on the overall health of the patient. The medication is most likely to cause problems in the case of overdose when it is taken by mouth or by IV, rather than applied to the skin or used vaginally [8].

High performance liquid chromatography [9–11], titrimetric method [12], and spectrophotometry [13] are the conventional methods reported for the determination of metronidazole in pharmaceutical sample. However, most of these methods are time-consuming, tedious, environmental nonfriendly, and expensive and require trained technicians [14].

On the contrary, electrochemical methods are promising alternatives for the determination of electroactive species, because of their inherent advantages of simplicity, ease of miniaturization, high sensitivity, and relatively low cost [15, 16]. Limited works have been reported on the electrochemical determination of metronidazole in pharmaceutical and clinical matrices using mercury electrode [17, 18], DNA-modified glassy carbon electrode [19], composite polymer membrane electrode [20], ultratrace graphite electrode (UTE) [21], activated glassy carbon electrode [22], and MWNT/glassy carbon electrode [23]. Although the reported methods are sensitive with detection to a nanomolar level, most of them have used mercury electrode which is environmental unfriendly, while the others have used expensive electrodes. Thus, the development of a simple, cost effective, and sensitive method is needed for the determination of metronidazole in pharmaceutical formulations. Thus, square wave voltammetric determination of metronidazole in tablet samples using carbon paste electrode (CPE) is presented in this study.

## 2. Experimental

### 2.1. Chemicals and Apparatus

Standard metronidazole and graphite powder (British Drug Houses Ltd., UK), metronidazole tablets of three brands, two Ethiopian brands (Addis Pharmaceuticals Factory (APF) and Ethiopian Pharmaceuticals factory (EPHARM)) and the third is an Indian brand, rabeprazole and tinidazole tablets (APF), omeprazole tablet (EPHARM), paraffin oil (Abron Chemicals Ltd.), phosphoric acid (EPHARMECOR), and boric acid, glacial acetic acid, and sodium hydroxide (Blulux Laboratories Ltd.) were used. All chemicals were of analytical grade that they were used without further purification.

A BAS 100B, electrochemical analyzer (Bioanalytical Systems (BAS), USA) with three-electrode system, carbon paste electrode as working electrode, platinum wire as auxiliary electrode, and Ag/AgCl as reference electrode, was used. Jenway model 3310 pH meter and an electronic balance (Denver Instrument) were used to measure the pH of the buffer solutions and the mass of different chemicals, respectively.

### 2.2. Procedure

#### 2.2.1. Preparation of Standard Solution

Britton Robinson buffer solution (BRB solution) in the pH range 2.0–12.0 was prepared from a mixture (0.1 M) of acetic acid, boric acid, and phosphoric acid. The pH of the solutions was adjusted using 1 M sodium hydroxide solution.

A stock solution of 5 mM standard metronidazole solution was prepared by dissolving 0.0856 g of metronidazole in 100 mL of distilled water. The required metronidazole working solutions were prepared by diluting the stock solution with the BRB solution of the appropriate pH.

#### 2.2.2. Pharmaceutical Tablet Sample Preparation

Metronidazole tablets (all labelled as 250 mg per tablet) of three brands, two of which are Ethiopian (Addis Pharmaceuticals Factory (APF) and Ethiopian Pharmaceuticals Factory (EPHARM)) and the third is an Indian factory (Aurobindo Pharmaceutical Industries), were collected from a pharmacy. Five tablet formulations of each brand were accurately weighed and ground using mortar and pestle. An adequate amount of this powder, corresponding to a stock solution of concentration 1 × 10^−2^ M, was weighed and transferred into a 100 mL flask and filled to the mark with distilled water. An intermediate tablet solution of 5 mM concentration was prepared from the tablet stock solution using distilled water as a solvent. After filtration, 35, 70, and 88 *μ*M sample solutions were prepared from the tablet stock solution using BRB solutions for each brand of metronidazole tablet.

#### 2.2.3. Preparation of Working Electrode

Carbon paste electrode was prepared by thoroughly mixing 1 g of graphite powder with paraffin oil in a ratio of 72% (w/w) graphite powder and 28% (w/w) paraffin oil [24]. The mixture was homogenized with mortar and pestle for 30 minutes and allowed to stand for 24 hrs. The homogenized paste was packed into the tip of a plastic tube of diameter 3.5 mm (chewing gum stick bought from ordinary shop). After copper wire was inserted from the backside of the plastic tube to provide electrical contact, the electrode was made ready for use after the surface of the electrode was smoothed manually against a smooth white paper until a shiny surface is emerged.

### 2.3. Method of Analysis

Cyclic voltammetry in the potential window +500 to −1200 mV was used for the investigation of the electrochemical behavior of standard metronidazole at carbon paste electrode. The effects of scan rate in the range of 10 to 250 mV/s and pH in the range 20 to 120 on the peak potential and peak current of metronidazole were also studied using cyclic voltammetry. For the quantitative determination of metronidazole using carbon paste as a working electrode, a square wave voltammetry in the range −100 to −1100 mV was employed. Linear calibration curve for the dependence of square wave peak current on the concentration of standard metronidazole was obtained. Moreover, the metronidazole content of different brands of metronidazole tablets was determined. Recovery results of spiked standard metronidazole in tablet solutions, interference study results, method detection limit, linear range, and precession of the results obtained were used to validate the applicability of the developed method for the determination of metronidazole in pharmaceutical formulations.

## 3. Results and Discussion

### 3.1. The Cyclic Voltammetric Investigation of Metronidazole at CPE

#### 3.1.1. Electrochemical Behavior of Metronidazole

**Figure 1:**
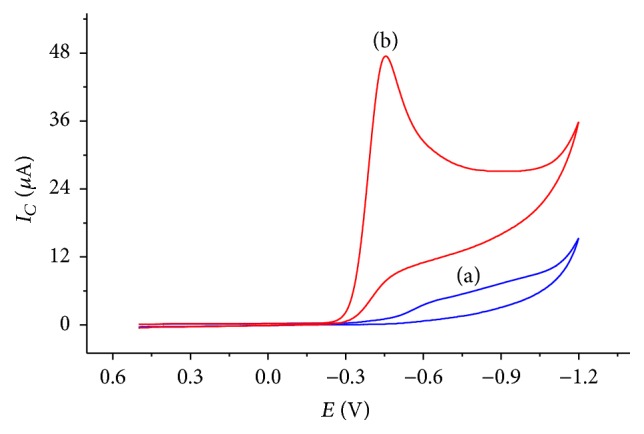
Cyclic voltammograms of CPE in BRB solution containing (a) no MTZ and (b) 1 mM MTZ at pH 2.0 and scan rate 100 mV/s.

Figure 1 presents the cyclic voltammograms of CPE in BRB solution (pH 2.0) in the presence and absence of 1 mM metronidazole. In the absence of metronidazole, a weak and broad reductive peak that appeared between −500 and –1050 mV was ascribed to the oxygen reduction (curve (a) of Figure 1). On the contrary, a well-defined, intensive, and sharp reductive peak centered at −450 mV (curve (b) of Figure 1) was observed in the presence of 1 mM metronidazole indicating an irreversible reduction of metronidazole at CPE.

#### 3.1.2. Effect of Scan Rate

**Figure 2:**
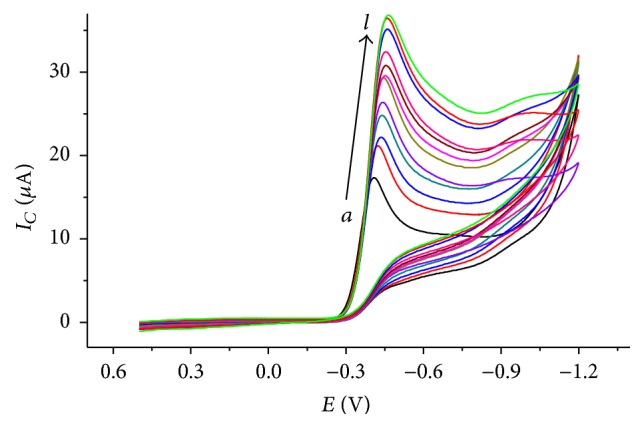
Cyclic voltammograms of 1 mM metronidazole in BRB solution (pH 2.0) using CPE at various scan rates (*a*–*l*: 10, 20, 40, 60, 80, 100, 125, 150, 175, 200, 225, and 250 mVs^−1^, resp.).

The effects of scan rate on the reduction peak current and peak potential of metronidazole were studied.  Figure 2 describes the cyclic voltammograms of 1 mM metronidazole at scan rate range of 10–250 mV/s. Cathodic peak potential shifted to a larger negative potential value confirming the irreversibility of the reduction reaction of MTZ at CPE [25].

In order to investigate whether the reduction process of metronidazole at CPE is predominantly diffusion controlled or adsorption controlled process, the correlation coefficients for the linear plots of the reductive peak current* versus* the scan rate and reductive peak current* versus *the square root of scan rate were compared (figure not shown). In contrast, a better correlation coefficient for the dependence of reductive peak current on the scan rate (*r* = 0.999) than on the square root of scan rate (*r* = 0.993) indicated that the reduction of metronidazole at CPE is governed by both surface-adsorption and diffusion kinetics though it is predominantly surface-confined kinetics [26].

Furthermore, the number of electrons transferred (*n*) for the surface-confined irreversible process was estimated employing the following equations [25, 27]:

(1) IPC=αnn2F2Γν2.718RT,

(2) EP−EP/2=1.85RTαnF V=0.048αn Vat  25°C,

(3) Γ=QnFA,

where *I*_*P*_ is peak current, *ν* is scan rate, Γ is surface concentration of the electroactive species in mol cm^−2^, *A* is electrochemical active electrode surface area in cm^2^, *α* is electron transfer coefficient, *R* is the universal gas constant (8.314 J K^−1^ mol^−1^), *T* is the Kelvin temperature, *F* is Faraday constant (9,6485 C mol^−1^), *E*_*P*_ is peak potential, *E*_*P*/2_ is half peak potential, and *Q* is the charge consumed in coulomb obtained by integrating the peak area. Taking the voltammogram at a scan rate of 100 mV in Figure 2, *αn* value estimated using (2) was calculated to be about 0.913. Substituting Γ term of (3) into (1), a new relation was obtained (4) from which the number of electrons transferred (*n*) in the rate determining step was calculated to be 3.77 (≈4) which is in agreement with earlier reported work [17]

(4) $$\begin{array}{c} n=\frac{2.718I_{P}RT}{\left(\alpha n\right)FQ\nu }. \end{array}$$

The value of *α* was then calculated to be 0.228, still confirming the irreversibility of the reduction of MTZ at CPE.

Plot of reductive peak potential (*E*_*PC*_) against the logarithm of scan rates (ln⁡*ν*) showed linear dependence with a linear regression equation and correlation coefficient of *E*_*PC*_/V = −0.37356 − 0.026157 ln⁡*ν*/Vs^−1^ and *r* = 0.999, respectively (figure not shown), from which the standard rate constant value for an irreversible reductive reaction was calculated using the following equation [28]:

(5) $$\begin{array}{c} E_{P}=E^{o}+\frac{RT}{\alpha nF}\ln\left(\;\frac{RTk_{0}}{\alpha nF}\;\right)-\frac{RT}{\alpha nF}\ln\nu , \end{array}$$

where *E*_*P*_ is the peak potential, *E* ^*o*^ is the formal potential, *α* is the electron transfer coefficient, *k*_0_ (s^−1^) is the electrochemical rate constant, and the other parameters have their usual meanings.

After calculating *E* ^*o*^ from the linear regression equation of the graph of *E*_*P*_ *versus ν* (figure not shown) [29], the value of *k*_0_ was calculated from the intercept of the plot of *E*_*P*_ *versus *ln⁡*ν* (figure not shown) to be 280.38 s^−1^

(6) $$\begin{array}{c} E^{o}+\frac{RT}{\alpha nF}\left[\;\ln\left(\;\frac{RTk_{0}}{\alpha nF}\;\right)\;\right]=-0.3736\;V. \end{array}$$

#### 3.1.3. Effect of Solution pH

**Figure 3:**
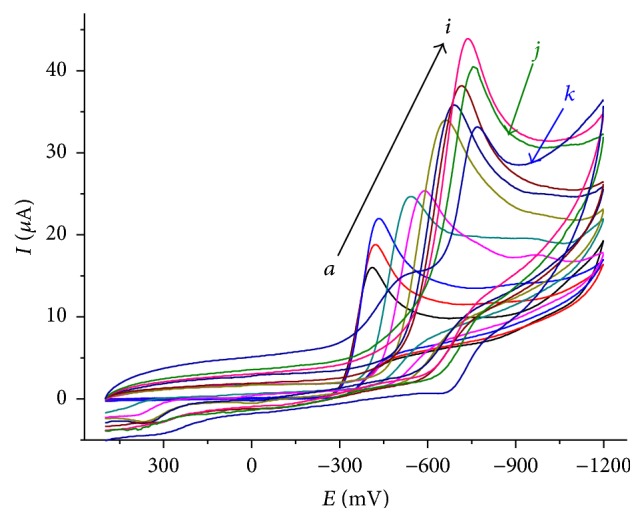
Cyclic voltammograms of 1 mM MTZ in BRB solution of different pH values (*a*–*k*: 2.0, 2.5, 3.5, 4.0, 4.5, 5.0, 6.0, 6.5, 7.0, 7.5, and 8.0, resp.). Scan rate: 100 mV/s.

The effect of pH on the reductive peak current and peak potential of metronidazole at CPE was further studied. BRB solutions with pH values varying from 2.0 to 12.0 were used to investigate its effect on the reduction of metronidazole at carbon paste electrode. The results revealed that voltammetric responses were strongly pH dependent in the acidic and neutral region in contrast to the alkaline medium which was in agreement with most of the electrochemical methods reported. A peak potential independent of pH in the alkaline medium could be attributed to the unavailability of protons that promote the reduction of MTZ at pHs larger than its pka value.  Figure 3 represents cyclic voltammograms of 1 mM MTZ in the region where its reduction is pH dependent (pHs 2.0–8.0). A well-defined irreversible cathodic peak was observed in the entire buffer system at the CPE.

**Figure 4:**
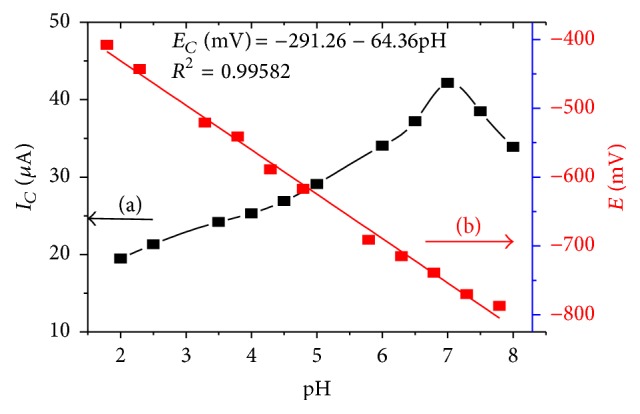
Plot of reductive (a) peak current and (b) peak potential* versus *pH for 1 mM MTZ in 0.1 M BRB solution of different pH values at CPE. Scan rate: 100 mV/s.

The effect of pH on the peak current in the studied pH range was investigated (Figure 4(a)). The cathodic peak current increased sharply from pH 2.0 to 7.0 beyond which it started to decrease. Thus, pH 7.0 was selected as the optimum pH for the subsequent experiments which is in agreement with previous works [16].

**Scheme 1:**
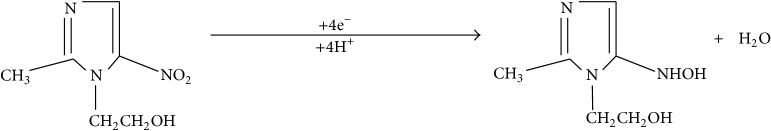
The proposed reaction mechanism of metronidazole at CPE.

With increasing solution pH up to 8.0, a peak potential shift in the negative potential direction was observed indicating the involvement of protons during the reduction reaction of metronidazole in acidic and neutral media (Figure 3). This trend was in agreement with reported works [16–18]. A linear dependence of peak potential on solution pH in the pH range 2.0–8.0 (Figure 4(b)) with a linear regression equation and correlation coefficient of *E*_*PC*_ (mV) = −291.26 − 64.36pH and *r* = 0.999, respectively, was observed. A slope of 64.36 mV/pH typically suggested that the number of protons taking part in the reaction is similar to the number of electrons that participated in the rate determining step. Hence, a reaction mechanism involving four electrons and four protons was proposed (Scheme 1).

### 3.2. Quantitative Determination of Metronidazole in Pharmaceutical Formulations

Square wave voltammetry (SWV), which is one of the most sensitive voltammetric techniques, was used for the quantitative determination of metronidazole in tablet samples.

**Figure 5:**
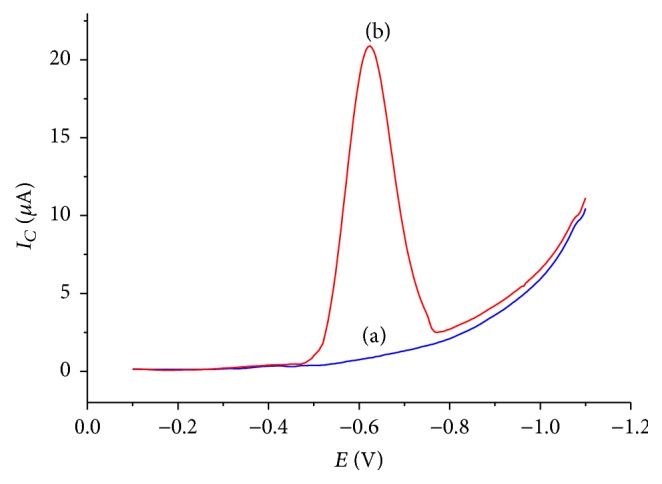
Square wave voltammograms of CPE in pH 7.0 BRB solution containing (a) no MTZ and (b) 1 mM MTZ.

Figure 5 presents the square wave voltammograms of CPE in pH 7.0 BRB solution containing no (a) and 1 mM metronidazole (b). As can be seen from the figure, no peak is observed at CPE in the buffer solution containing no MTZ (curve (a) of Figure 5). In contrast, the same working electrode in pH 7.0 BRB solution containing 1 mM MTZ resulted in an enhanced reductive peak (curve (b) of Figure 5).

#### 3.2.1. Linear Range and Limit of Detection

**Figure 6:**
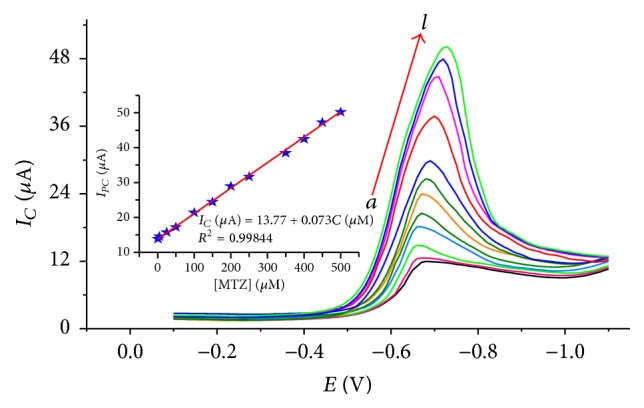
Square wave voltammograms of CPE (corrected for background) in pH 7.0 BRB solution containing MTZ of various concentrations (*a*–*l*: 1, 5, 25, 50, 100, 150, 200, 250, 350, 400, 450, and 500 *μ*M, resp.). Inset: calibration curve of peak current* versus *concentration of MTZ. Experimental conditions: square wave frequency 30 Hz, square wave amplitude 50 mV, step potential 10 mV, *E*_acc_  −500 mV, and *t*_acc_ 25 s.

Under the optimum experimental conditions (pH, accumulation potential (*E*_acc_), accumulation time (*t*_acc_), SWV frequency, amplitude, and step potential of 7.0, −500 mV, 25 s, 30 Hz, 50 mV, and 10 mV, resp.), the dependence of square wave voltammetric peak current on the concentration of MTZ and the inherited sensitivity of the method were investigated in the range 1 × 10^−6^–5 × 10^−4^ M.  Figure 6 shows square wave voltammograms of various concentrations of MTZ corrected for background.

Inset of Figure 6 presents the plot of the reductive peak current as a function of the concentration of MTZ. The limit of detection (LOD) and limit of quantification (LOQ) calculated using (7) and (8) were found to be 2.97 × 10^−7^ and 9.91 × 10^−7^, respectively:

(7) LOD=3Sm,

(8) LOQ=10Sm,

where *S* is the standard deviation for the blank (*n* = 8) and *m* is the slope of the calibration curve.

Compared to the previously reported works which have used expensive electrode materials, CPE which is the cheapest carbon based material revealed a comparable LOD.

#### 3.2.2. Real Sample, Recovery, and Interference Analyses

The selectivity and the accuracy and hence the validity of the carbon paste electrode for the determination of MTZ in real samples were demonstrated by evaluating its application for the determination of MTZ content in some pharmaceutical tablets. Briefly, five tablets from each brand (APF, EPHARM, and Indian) were weighed and powdered. For each brand of tablet, an amount corresponding to a stock solution of 0.01 M concentration was weighed and transferred into 100 mL flask and then completed to the volume with pH 7.0 BRB solution. Finally, 35, 70, and 88 *μ*M tablet sample solutions were prepared from the corresponding stock solution. Square wave voltammograms were recorded (figure not shown) following the outlined voltammetric procedure and optimized conditions as described earlier. Mean of triplicate measurements was taken for the determination of metronidazole in these samples.

The results for different concentrations of the three brands of tablets are summarized in Table 1. The tablet formulations for the collected brands being all 250 mg of MTZ per tablet, the amount of MTZ detected relative to the expected value according to the label in the APF, EPHARM, and Indian brand tablets were about 90.139%, 96.235%, and 93.544%, respectively. Detected values lower than the prescribed value may be due to the possible mass loss of MTZ during preparation or sort of degradation during storage, otherwise originally lower levels of MTZ in the tablets.

The square wave voltammograms for 35 *μ*M tablet samples of each brand tablet, each spiked with the same amount of standard MTZ (100 *μ*M), were recorded (figures not shown). As can be observed from Table 2, recovery results in the range of 95.374% to 97.331% confirmed the potential applicability of the developed method for MTZ analyses in real samples. Relative standard deviation (RSD) of 2.439 which is comparable to elsewhere reported work using UTGE [18] showed the reliability of our result.

To further elaborate the potential applicability of the method, the selectivity of the method for MTZ in the presence of potential interferents was studied. For the interference studies, drugs which could be present in the MTZ tablet (rabeprazole and omeprazole) or have structural similarities with MTZ (tinidazole) were selected. The effect of each selected potential interferent was investigated at various concentrations of the interferents (figure not shown) added to 35 *μ*M MTZ. As can be seen from Table 3, the presence of different concentrations of rabeprazole and omeprazole with a fixed concentration of metronidazole did not significantly affect the peak current response for the MTZ and the change in peak current was less than 5%. However, the presence of tinidazole in whatever amount showed positive interference as in the reported works [11].

## 4. Conclusion

Cyclic voltammetric investigation of metronidazole at CPE revealed an irreversible reduction peak over the studied potential window. While the peak potential shift with scan rate confirmed the irreversibility of the reaction, peak potential shift with pH also indicated the involvement of protons in the reduction process. The calculated kinetic parameters of the reduction of metronidazole at carbon paste electrode are found to be in agreement with the proposed reaction mechanism in literature. Under the optimized solution pH, SWV, and accumulation parameters, carbon paste electrode showed relatively wide linear range with comparable LOD, LOQ, recovery, and selectivity relative to the previously reported works which have used expensive electrodes.

Hence, the developed electrochemical method using the cheapest carbon based electrode can be used as an alternative method for the determination of metronidazole even in a complex matrix system like pharmaceutical formulations.

## Figures and Tables

**Table 1:** Amount of MTZ detected in three brands of drug samples using the developed method.

Tablet	Solution	Expected/*μ*M	Detected^*∗*^		Labelled value (mg/tablet)	Measured%
			/*μ*M	/(mg/tablet)		
APF	a	35	28.789	205.640	250	82.256
	b	70	63.125	225.445	250	90.178
	c	88	86.225	244.957	250	97.983

EPHARM	a	35	32.573	232.665	250	93.066
	b	70	67.178	239.919	250	95.968
	c	88	87.709	249.175	250	99.670

Indian	a	35	32.774	234.103	250	93.641
	b	70	63.308	226.099	250	90.440
	c	88	84.965	241.378	250	96.551

^*∗*^Mean of triplicate measurements.

**Table 2:** Percentage recovery of MTZ from pharmaceutical tablets.

Tablet	Present MTZ/mg	Added MTZ/mg	Expected MTZ/mg	Found/mg^*∗*^	Recovery (%) ± % RSD
APF	0.091	0.086	0.177	0.173 ± 0.0042	95.604 ± 2.439
EPHARM	0.112	0.086	0.198	0.195 ± 0.0042	97.321 ± 2.154
Indian	0.114	0.086	0.200	0.195 ± 0.0040	95.614 ± 2.054

^*∗*^Mean of double measurements.

**Table 3:** Interference study of MTZ with different concentrations of rabeprazole, omeprazole, and tinidazole.

Interferent	Concentration in *μ*M of the interferent added to 35/*μ*M MTZ	Recorded signal (*I*_*P*_/*μ*A)	Signal change (%)
Rabeprazole	0	**38.19**	—
	117	37.68	1.335
	233	36.64	4.059
	350	36.53	4.347

Omeprazole	0	**38.19**	—
	117	37.19	2.618
	233	36.64	4.059
	350	36.53	4.347

Tinidazole	0	**38.19**	—
	117	40.77	6.756
	233	41.79	9.426
	350	44.23	15.816
